# Health-economic evaluation of a novel personalized digital avatar-based anemia management software in hemodialysis patients

**DOI:** 10.1038/s41746-025-02209-6

**Published:** 2026-01-03

**Authors:** Afschin Gandjour, Dana Kendzia, Kevin Ho, Doris H. Fuertinger, Carsten Hornig, Christian Apel, Jovana Petrovic Vorkapic

**Affiliations:** 1 Frankfurt School of Finance & Management, Frankfurt, Germany; 2 Market Access, Health Economics & Political Affairs, Fresenius Medical Care Deutschland GmbH, Bad Homburg, Germany; 3 Fresenius Medical Care North America, Waltham, MA USA; 4 Renal Research Institute, New York, NY USA

**Keywords:** Diseases, Health care, Medical research, Nephrology

## Abstract

This study aimed to evaluate the cost-effectiveness and financial impact of an anemia management tool (AMT)—a software system that uses real-time blood volume and hemoglobin monitoring data—for adult patients receiving in-center hemodialysis (HD) in the United States. A Markov cohort model was developed to estimate lifetime costs and health outcomes for 1000 in-center HD patients with and without use of AMT. Clinical input parameters, including hemoglobin stability and dose reduction of erythropoiesis-stimulating agents (ESAs), were derived from a randomized controlled trial. The net monetary benefit (NMB) was calculated from the Medicare perspective, while a net financial impact analysis (NFIA) estimated provider-level savings based on ESA dose reductions, Quality Incentive Program (QIP)-related payment adjustments, and implementation costs. From the Medicare perspective, AMT yielded a positive NMB of $8419 per patient over a lifetime and remained cost-effective at a threshold of $2443 per patient per year. The NFIA showed an annual per-patient profit of $218. For a dialysis facility with 70 patients, this corresponds to an annual profit of $15,251. In conclusion, AMT is cost-effective from the Medicare perspective and financially beneficial for providers. Broader adoption may be supported by value-based reimbursement mechanisms and risk-sharing agreements to address residual uncertainties.

## Introduction

Chronic kidney disease (CKD) is categorized into five stages based on kidney function^1^. Stage 5, also referred to as end-stage kidney disease (ESKD), is typically treated with kidney replacement therapies such as dialysis or transplantation and affects more than 800,000 patients in the United States (U.S.)^2^. Over 90% of dialysis-dependent patients experience anemia^3^, which significantly reduces their quality of life (QoL) and is associated with numerous symptoms and complications^4^. In CKD patients, anemia is usually managed with iron replacement therapy and erythropoiesis-stimulating agents (ESAs). Administering ESAs to maintain higher Hb levels may increase the risk of death and serious cardiovascular events, including stroke, myocardial infarction, and heart failure^5^. The KDIGO Clinical Practice Guideline for Anemia in Chronic Kidney Disease (2012) recommends that ESAs not be used to maintain hemoglobin (Hb) concentrations above 11.5 g/dL (115 g/L) in adult patients with CKD. In agreement with this recommendation, the FDA^6^ warns that using ESAs to target Hb >11 g/dL increases the risk of serious cardiovascular events (death, MI, stroke, thromboembolism) and has not shown additional benefit; labels advise using the lowest ESA dose sufficient to reduce RBC transfusions and reducing or interrupting the dose as Hb approaches/exceeds 11 g/dL.

Moreover, large observational studies in the hemodialysis population (e.g., ref. ^7^) have shown that ESA-based anemia management is commonly associated with Hb variability, resulting in frequent transient excursions outside the target range—even when individual or population-level mean Hb values remain within it. Increased Hb variability, however, is associated with poor clinical outcomes, including higher mortality^8^. Therefore, careful and individualized consideration of the risks and benefits is essential when managing anemia in CKD patients^4^.

Anemia inSights™ (anemia management tool; AMT) is a clinical decision support software that builds on established and well-understood physiological and pharmacokinetic processes. The system comprises a comprehensive physiology-based model of erythropoiesis and erythrocyte dynamics^9,10^ that estimates patient-specific key physiological characteristics, such as red blood cell lifespan. For each patient, the system creates a set of personalized models (i.e., digital twin/patient avatar) utilizing routine clinical data (sex, height, recent body weights, Hb levels, and ESA doses)^11^. A model predictive controller^12^ processes the models’ predictions of individual Hb trajectories to provide fully personalized dosing recommendations for methoxy polyethylene glycol-epoetin beta (MIRCERA®), which serves as the exclusive ESA. The software is specifically designed to achieve and maintain Hb levels within narrow target ranges using ESA efficiently.

AMT is used alongside Crit-Line® monitoring (CLM) (Fresenius Medical Care, Bad Homburg, Germany), an intradialytic relative blood volume (RBV) monitoring device methodology based on the continuous measurement of hematocrit levels. Crit-Line utilizes optical sensors placed in the arterial bloodline to measure hematocrit, a marker of hemoconcentration^13,14^.

A recently published randomized controlled trial (RCT) by Fuertinger et al.^15^ compared AMT to the standard of care in hemodialysis (HD) patients over a 26-week period. In the trial, the Hb target was set at 10–11 g/dL. Patients in the AMT group, who received ESA dose recommendations from the software, demonstrated improved Hb stability within the target range. Target attainment in the intervention group increased to 47%, while the standard-of-care group showed no change from baseline^15^. Additionally, Hb variability decreased in the AMT group (45%) compared to the standard-of-care group (82%), and ESA dose usage was reduced by approximately 25% in the AMT group^15^. Clinician adherence to AMT was high: of 561 ESA dose recommendations, only 31 were overridden, with six additional deviations due to logistical constraints.

While the effectiveness of AMT has thus been demonstrated, its cost-effectiveness and overall value have yet to be established. This study aimed to evaluate the cost-effectiveness and healthcare value of implementing AMT in adult patients undergoing in-center HD in the U.S. from the Medicare payer perspective, and to assess the net financial impact (NFIA) of AMT from the perspective of a large dialysis provider.

## Results

The projected hazard ratio (HR) for mortality with AMT was 0.983 (range: 0.974–0.997), based on modeled relationships between hemoglobin variability and mortality risk, as described in the Methods section. The annual reduction in the hospitalization rate was estimated at 0.13, meaning 13 hospitalizations per 100 patients were avoided each year. In general, AMT increases expenditure when patients experience reduced mortality but decreases expenditure when hospitalization rates decline.

Table 1 shows that the cost-utility ratio for treating HD patients without AMT, compared to no treatment, is $152,000 per quality-adjusted life-year (QALY) gained. This value reflects Medicare’s willingness to invest in the treatment of ESKD patients. Assuming zero costs of AMT for Medicare, AMT dominates the no-AMT scenario, resulting in lower lifetime health expenditures and a QALY gain of 0.04 (Table 1). Providing AMT to all patients aged 65 and older receiving in-center HD in the U.S. is projected to save $94 million annually, equivalent to 1% of expenditures for in-center HD patients.

**Table 1:** Costs, effects, and cost-effectiveness of the anemia management tool (AMT) compared to the standard of care

Treatment	Costs	Life years	QALYs	Incremental costs per life year gained	Incremental costs per QALY gained
AMT	405,910	3.97	2.74	−79,751	−115,581
Standard of care	410,106	3.92	2.70	104,654	151,672

All costs are in U.S. dollars.

*QALY* quality-adjusted life year.

The NMB of AMT from the Medicare payer perspective is estimated at $8419 per patient over a lifetime, or $2443 per patient per year. This annual value represents the maximum price that could be charged for AMT while maintaining cost-effectiveness.

The NFIA indicates a profit of $218 per patient per year, based on ESA savings, additional revenue from avoiding a 0.12% payment reduction under the End-Stage Renal Disease Quality Incentive Program (ESRD QIP) ($49.71 per patient per year), and accounting for the combined cost of CLM and AMT ($347 per patient per year). For a dialysis facility with 70 patients, this translates to an annual profit of $15,251.

**Fig. 1:**
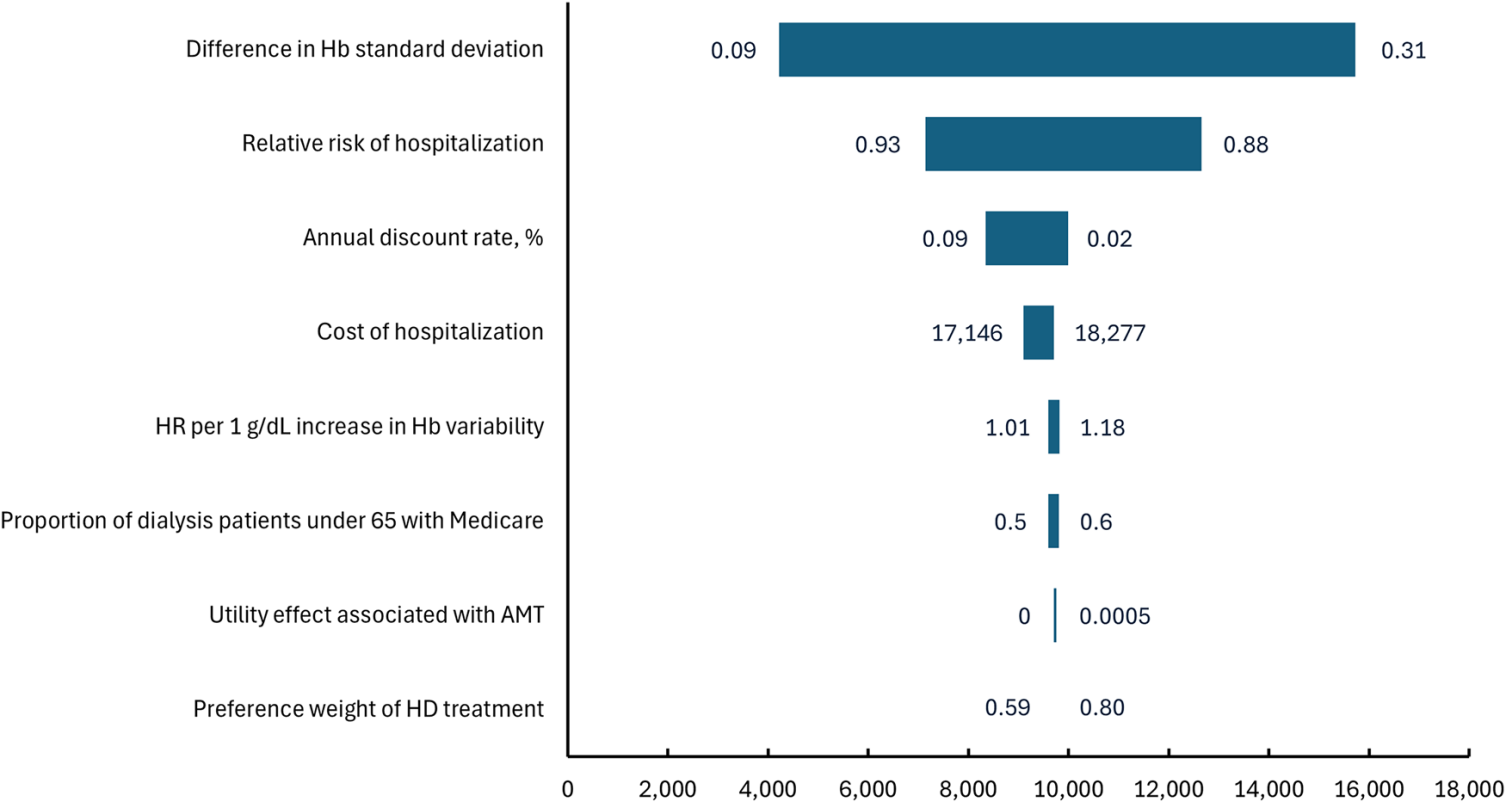
Tornado diagram illustrating the results of the one-way sensitivity analysis on the net monetary benefit of the anemia management tool (AMT). Variables are ordered by impact on the net monetary benefit of AMT. Hb hemoglobin, HR hazard ratio, HD hemodialysis.

**Fig. 2:**
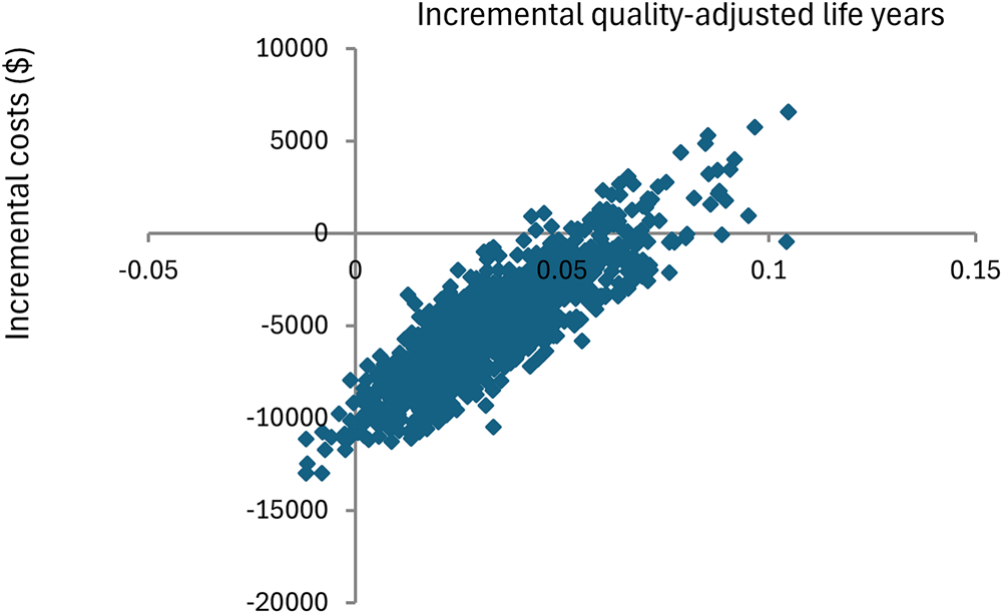
Scatter plot of results from the Monte Carlo simulation.

In the one-way sensitivity analysis, the reduction in Hb standard deviation achieved by AMT was the single most influential factor in lifetime NMB (Fig. 1). Parameters were varied to their prespecified low/high values while others were held constant; bars show how each change shifts NMB away from the base-case value. In the probabilistic sensitivity analysis (PSA) with 1000 Monte Carlo iterations, all iterations produced a non-negative NMB at the willingness-to-pay threshold defined in the Methods, indicating that AMT was consistently cost-effective relative to no AMT (Fig. 2). The simulated results were stable, with a 95% CI for lifetime NMB of $7280 to $13,121.

**Fig. 3:**
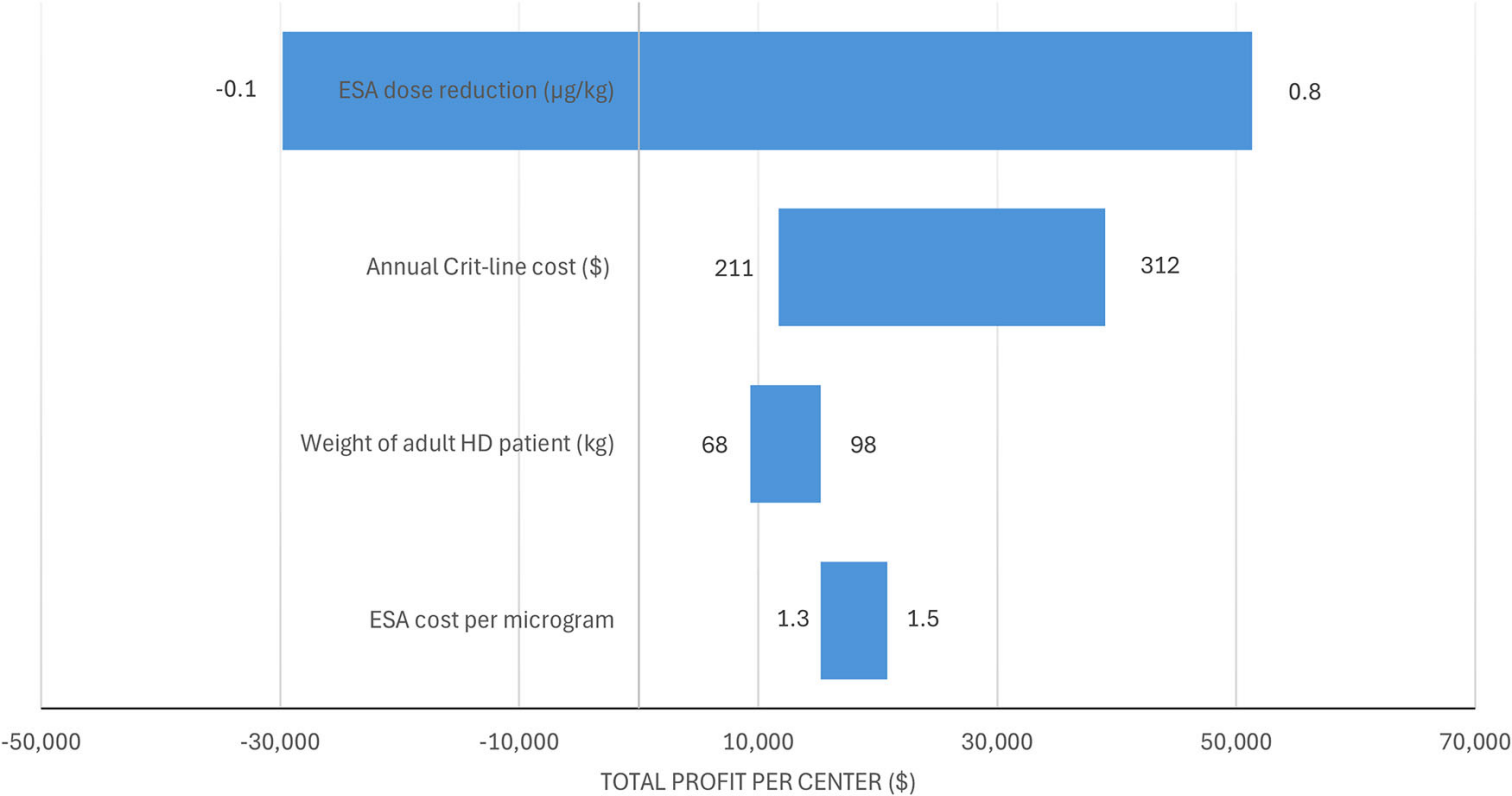
Tornado diagram demonstrating the results of the one-way sensitivity analysis on the annual profit of a dialysis center. Variables are ordered by impact on the annual profit of a dialysis center. ESA erythropoiesis-stimulating agent, HD hemodialysis.

The variable with the largest impact on the annual profit of a dialysis center was the reduction in ESA dose achieved by AMT (Fig. 3). When considering only the cost of CLM consumables and AMT computations, the annual profit increases to $20,492. If only the computational cost of AMT is considered, the annual profit rises to $39,002.

## Discussion

Based on reductions in mortality and hospitalization rates, AMT has been shown to be cost-effective compared to the standard of care in the Medicare population. This result was confirmed through Monte Carlo simulation (MCS), which demonstrated 100% cost-effective iterations. The NMB analysis indicates that the maximum price Medicare could pay for AMT while maintaining cost-effectiveness is $2443 per patient per year.

From the perspective of a large dialysis provider, AMT could yield an annual profit of approximately $15,000 for a dialysis facility with 70 patients. This average number of patients—and hence the annual profit—varies depending on geographic location, with urban centers typically serving more patients than rural ones^16^. The profit estimate is based on savings on ESAs and increased revenue from avoiding payment reductions under the ESRD Quality Incentive Program. This finding is particularly relevant because under the ESRD Prospective Payment System, any reductions in ESA use provide a direct financial benefit to dialysis clinics rather than to Medicare. Thus, the economic incentive for providers to adopt AMT is clear and immediate.

Additional benefits of AMT, beyond anemia management, were not included in this calculation. As CLM serves as the backbone of AMT, it can also be used to optimize fluid management during dialysis sessions, thereby reducing fluid overload and intradialytic hypotension episodes^17,18^.

Our findings that an AI-guided anemia management strategy yields economic value are directionally consistent with a recent German analysis of the Anemia Control Model (a distinct system), which reported that ACM results in both increased QALYs and reduced costs compared to standard care^19^. Also in line with our findings, a cost-effectiveness model by Quon et al.^20^ using U.S. data suggests that maintaining Hb levels between 10–11 g/dL results in reduced hospitalizations and increased QALYs. However, the cost-effectiveness of using ESAs in dialysis patients varies significantly in the literature due to differences in modeling assumptions. There is ongoing debate about the economic acceptability of targeting higher Hb levels^21^.

Several limitations of this study should be noted. First, the RCT by Fuertinger et al.^15^ recruited participants exclusively from Fresenius Kidney Care dialysis clinics in the U.S., which may limit generalizability. Care pathways, staffing models, supply contracts (e.g., ESA products), electronic health record (EHR) integration, and quality-improvement infrastructure can differ in non-FKC U.S. providers (independents, hospital-based units) and in other countries. Because our base case adopts a U.S. Medicare ESRD prospective payment system (PPS) perspective—with ESA, iron, and most labs paid from a fixed bundle—the model’s cost offsets from reduced drug use accrue to the payer and/or facility depending on local contracts. In fee-for-service or less tightly bundled systems, the direction and magnitude of budget impact could differ (e.g., facility margins may be less sensitive to ESA reductions, whereas payer drug spend may be more sensitive). Differences in anemia targets, lab cadence, product choice, and adherence to digital decision support could also shift both effectiveness and costs. We therefore recommend interpreting results as most applicable to large, integrated U.S. dialysis networks operating under Medicare PPS, with sensitivity analyses illustrating how outcomes might change in alternative settings.

Second, the impact on mortality and hospitalizations was not directly assessed in the RCT but projected based on secondary data. Third, the study underestimated potential savings from AMT by excluding reductions in adverse events (AEs) associated with lower ESA doses. These AEs include thromboembolic events such as arterial embolism, thrombophlebitis, and venous or limb thrombosis. However, predicting the exact reduction in AE incidence in response to a given ESA dose reduction remains challenging. Fourth, our results are most applicable to settings where MIRCERA is the predominant ESA and AMT is configured for its pharmacokinetic/pharmacodynamic (PK/PD) profile. MIRCERA use has grown in recent years, but is not universal^22^. If other ESAs (e.g., shorter-acting epoetins or darbepoetin) are commonly used, effects on Hb stability should remain directionally similar if the controller is re-parameterized to the alternative ESA’s PK/PD; however, the magnitude and operational performance may differ (dose-titration granularity, dosing interval, nurse/physician workflow). Economically, provider margins may also change with different ESA acquisition prices/rebates and with any differences in dose requirements.

This study demonstrates that AMT offers considerable health-economic value for hemodialysis patients. By stabilizing hemoglobin levels and reducing variability, AMT is projected to decrease hospitalization rates and improve mortality outcomes, contributing to its cost-effectiveness compared to the standard of care in the U.S. Medicare population. The findings indicate a maximum price of $2443 per patient per year for AMT to remain cost-effective under Medicare’s perspective, while offering substantial financial benefits for large dialysis providers through ESA dose reductions and quality incentive adjustments.

In addition, AMT implementation may yield notable time savings for both nurses and physicians (nephrologists). In a typical dialysis clinic, anemia management is overseen by a designated anemia manager—often a nurse, charge nurse, or nurse manager—who is responsible for reviewing laboratory results and adjusting ESA doses based on clinic- or protocol-driven algorithms. Anemia outcomes are typically reviewed monthly during the medical director’s meeting, where individual patients may be discussed in greater detail.

Without AMT, anemia managers are tasked with ESA administration, manual dose adjustments, and ongoing monitoring of patient responses to optimize Hb levels. These responsibilities often involve extensive manual chart reviews, frequent communication with prescribing physicians, and blood draws for Hb monitoring. Physicians, in turn, review laboratory trends, adjust ESA dosing, and prescribe iron supplementation as needed. If AMT reduces the frequency of ESA or MIRCERA dose changes over time—by improving dosing precision or stability—it may directly decrease the time spent on lab reviews and dose adjustments, particularly for anemia managers.

Future research should prioritize broader evaluations of AMT across diverse dialysis centers and patient populations while accounting for indirect benefits, such as reduced adverse events from ESA dose adjustments. In addition, future studies should examine the impact of AMT on clinical workflow and staff workload, as these operational factors may influence adoption and scalability. Given the uncertainties associated with hospitalizations and mortality, risk-sharing agreements between Medicare and the manufacturer could serve as an effective mechanism to manage these risks and ensure equitable access to AMT. Performance-based risk-sharing agreements (PBRSAs), already explored in other healthcare settings, offer a valuable framework for addressing cost-effectiveness uncertainties while aligning incentives between stakeholders^23^.

Under Medicare’s current reimbursement structure, the integration of PBRSAs for AMT would require adaptation within established frameworks, such as the ESRD Prospective Payment System and the Comprehensive ESRD Care (CEC) model. PBRSAs would entail reimbursing AMT based on predefined clinical and economic outcomes. Data collection during early adoption could provide critical evidence on these outcomes, enabling Medicare to reassess coverage and reimbursement levels periodically.

Implementation would hinge on several factors. First, clear metrics must be defined that are actionable, attributable to the AMT, and measurable using existing data infrastructure in dialysis centers^23^. Appropriate examples include Hb variability, hospital admissions, mortality, and ESA usage. Second, a collaborative approach among payers, providers, and manufacturers is essential. Manufacturers could share financial risks with providers by adjusting pricing based on outcome achievements—for example, by offering rebates, deferred payments, or pay-for-performance models. This would allow providers to adopt AMT without incurring significant upfront financial burdens.

In parallel, payers and manufacturers could establish broader agreements to manage reimbursement risk based on clinical and economic outcomes. The structure of these payer–manufacturer agreements could draw from the experience of PBRSAs in kidney care and other areas, such as the United Kingdom’s Cancer Drugs Fund or managed entry agreements in Europe^23^. For instance, temporary reimbursement at a reduced price could be provided until sufficient real-world data are collected to demonstrate AMT value. If outcomes fall short of agreed-upon benchmarks, the manufacturer could face price reductions or rebates. Conversely, exceeding benchmarks could lead to bonus payments, fostering shared accountability and innovation.

Challenges to implementing PBRSAs under Medicare include ensuring robust data collection processes, overcoming administrative burdens, and addressing the fragmented nature of the U.S. healthcare system. For PBRSAs to succeed, Medicare might need to establish a dedicated framework, potentially through the Center for Medicare and Medicaid Innovation (CMMI), which already supports innovative payment models like the CEC model.

Ultimately, PBRSAs could bridge the gap between innovation and affordability, providing Medicare beneficiaries with access to cutting-edge technologies like AMT while ensuring fiscal responsibility. Expanding the scope of these agreements in kidney care has the potential to redefine how novel treatments are introduced and evaluated, benefiting patients, providers, and payers alike. However, further dialogue among stakeholders and pilot programs will be necessary to refine these agreements and demonstrate their feasibility on a broader scale.

In conclusion, this study suggests that AMT represents a cost-effective and financially viable innovation in anemia management for adult patients receiving in-center hemodialysis in the United States. From the Medicare payer perspective, AMT demonstrated a favorable net monetary benefit, primarily driven by reduced hospitalization rates and improved hemoglobin stability. The system remained cost-effective at a maximum price of $2443 per patient per year. Simultaneously, the net financial impact analysis showed that AMT could generate meaningful operational savings and revenue gains for dialysis providers through ESA dose reductions and enhanced performance under the ESRD Quality Incentive Program.

## Methods

In this study, we conducted a cost-utility analysis to evaluate health benefits from the perspective of the U.S. Medicare payer. Quality-adjusted life-years (QALYs) were used as the measure of health outcomes. QALYs are a standardized metric that facilitates comparisons across various medical interventions for different diseases by combining both the length and quality of life. They quantify individual preferences for different health states on a scale typically ranging from 0 (representing death) to 1.0 (indicating perfect health), with the possibility of including negative values to represent health states perceived as worse than death.

The analysis was conducted over the remaining lifespan of patients, comparing the use of AMT to its absence, with the latter representing the current standard of care in the U.S. To evaluate the economic impact of AMT, we calculated the net monetary benefit (NMB) using the following equation^24^:

1 $$NMB=\lambda \,\cdot \,\Delta E-\Delta C,$$

where $\lambda$ represents the maximum willingness-to-pay (WTP), $\Delta C$ denotes incremental costs, and $\Delta E$ signifies incremental effects. The WTP threshold was proxied using the cost-effectiveness of HD versus no kidney replacement therapy (KRT), which treats the comparator as having zero costs and zero benefits; this yields an intentionally conservative (lower) WTP estimate^25^. In real-world practice, patients who forgo dialysis typically receive conservative kidney management/palliative care, incurring some costs and yielding non-zero survival and health-related quality of life. Under this more realistic comparator (HD vs. conservative/palliative management), incremental costs are lower than in the ‘no KRT’ comparison, but incremental QALYs are also smaller, and typically by a larger proportion, leading to a higher ICER for HD—and thus implying a higher WTP threshold than our conservative proxy. Additionally, we estimated an annual NMB by dividing the total NMB by the remaining life expectancy with AMT.

Our analysis focused on average representatives of the Medicare population, regardless of their prior duration of dialysis therapy. We assumed continuous use of AMT throughout the patient’s remaining lifetime, with a constant effect associated with its implementation. All calculations were performed using Microsoft Excel (Microsoft Corporation, Redmond, WA, USA).

We developed a Markov (cohort) state transition model to simulate the outcomes and costs of 1000 in-center HD patients receiving AMT and 1000 patients receiving standard care without AMT, projecting results over the patients’ lifetimes. The model included two health states: “alive” and “dead,” with all patients initially entering the “alive” state. Within this state, we modeled the occurrence of hospitalizations.

We did not partition the alive state into “Hb in target (10–11 g/dL)” versus “Hb out of target” in the base case because the available evidence does not show a meaningful utility difference between ESA-treated dialysis patients inside vs. outside this window^26^. Accordingly, the base case assumes no direct utility effect of time in range. To test a face-valid alternative—that smoother Hb confers a modest HRQoL benefit—we ran a scenario with a small per-in-range utility premium.

Baseline characteristics at the start of the simulation were based on the average Medicare HD population, including age and other patient characteristics, as reported by the USRDS^2^.

Patients could transition to the “dead” state at any time, but did not switch to an alternative treatment arm. During each cycle, patients accrued QALYs and costs, with a cycle length of one year for the two health states. We applied the life-table method^27^, assuming that transition events occurred, on average, halfway through each 12-month cycle. The simulation continued until fewer than 0.1% of patients remained alive in both arms.

Key modeling assumptions are summarized in Box 1.

The budget impact analysis (BIA) was conducted from the perspective of the U.S. Medicare payer and evaluated the effects of AMT on mortality and hospitalizations. Savings from ESA use were excluded from the analysis because Medicare reimburses dialysis centers through a bundled payment under the End-Stage Renal Disease (ESRD) Prospective Payment System (PPS), which includes the costs of ESAs. This bundled payment provides dialysis centers with a fixed amount per treatment, regardless of the actual quantity or cost of ESAs used. As a result, any reductions in ESA use financially benefit dialysis centers rather than Medicare directly.

The budget impact was projected to increase with reductions in mortality and decrease with reductions in hospitalizations. The time horizon for the BIA was 1 year.

To estimate the budget impact, we developed an incidence-based lifetime cohort model using the Markov modeling approach. This model focused on the incidence of new-onset HD cases. The annual budget impact for this incident cohort was calculated by multiplying the number of incident cases by the estimated lifetime costs per case.

The NFIA was conducted from the perspective of a large dialysis provider, such as DaVita or Fresenius Kidney Care, and focused on the net financial outcome, accounting for both costs and revenues related to AMT.

Since Medicare reimburses dialysis services through a bundled payment, savings from reduced ESA use directly benefit providers. The calculation of ESA savings began by determining the difference in the mean ESA dose between the intervention and control groups, expressed in micrograms per kilogram per 30 days. This difference was multiplied by the average patient weight to estimate the annual reduction in ESA usage per patient. The cost of ESA per microgram, which varies based on contract pricing, was then applied to calculate annual cost savings per patient.

In addition, a reduction in hospital admissions may financially benefit dialysis facilities through the Centers for Medicare & Medicaid Services (CMS) Quality Incentive Program (QIP)^28^. By achieving lower-than-expected 30-day readmission rates, facilities can avoid payment penalties, thereby potentially increasing their revenue. This aspect was also considered in the NFIA.

To complete the NFIA and calculate the net profit, we subtracted the costs of implementing CLM (device and/or consumables) and AMT from the total financial benefits.

Clinical inputs (e.g., Hb time-in-range, reduction in Hb standard deviation (SD), ESA dose change) were taken from the RCT by Fuertinger et al.^15^ (see Tables 2 and 3 for input data). The Fuertinger et al. RCT enrolled adult, thrice-weekly in-center hemodialysis patients treated across five Fresenius Kidney Care clinics (NY, CT, CA), using a standardized SOC anemia protocol—a large-network setting that mirrors how most U.S. (Medicare) HD care is delivered. The trial’s Hb target (10–11 g/dL) aligns with U.S. guideline recommendations and FDA cautions, reinforcing clinical relevance to Medicare practice. Patients received MIRCERA® exclusively; while not universal, its use has increased in U.S. HD^22^, supporting applicability to many Medicare HD facilities.

**Table 2:** Input values and distributions used in the base case cost-effectiveness analysis and sensitivity analysis

Input	Base case value (range)	PSA distribution	Parameters	Reference
*Epidemiological data*				
Incidence of patients with in-center HD				Figure 1.9 in USRDS^2^
Age 18−44	12,252	Fixed		
Age 45−64	40,998	Fixed		
Age 65−74	31,775	Fixed		
Age 75+	27,855	Fixed		
Proportion of dialysis patients under 65 with Medicare	0.55 (0.50–0.60)	Uniform	min = 0.50; max = 0.60	^33,34^
*Clinical data*				
All-cause mortality of hemodialysis p. a.				Figure 6.1b in USRDS^2^
Age 18−44	0.0963	Fixed		
Age 45−64	0.1536	Fixed		
Age 65–74	0.2326	Fixed		
Age 75+	0.3303	Fixed		
Reduction in Hb standard deviation by AMT, g/dl	0.2 (95% CI, 0.04−0.3)	Beta-PERT(α,β)	*α* = 3.462, *β* = 2.538	^15^
Increase in % of Hb values within the target with AMT	10			^15^
Hazard ratio for all-cause mortality per 1 g/dL increase in Hb variability	1.09 (95% CI, 1.01−1.18)	Lognormal(μ,σ)	ln-μ = 0.086, ln-σ = 0.040	^8^
All-cause annual hospitalization rate in adult hemodialysis patients (Medicare FFS)	1.49	Fixed		Figure 5.1a in USRDS^2^
Relative risk of hospitalizations for patients within the 10–11 g/dL range	0.91 (95% CI, 0.88−0.93)	Lognormal(μ,σ)	ln-μ = −0.092, ln-σ = 0.014	^7^
*Economic data*				
Preference weight of HD treatment	0.69 (95% CI, 0.59−0.80)	Beta(α,β)	*α* = 50.737, *β* = 22.795	^41^
Costs of an HD patient per annum				Figure 9.12a in USRDS^2^
Age 65–74	102,206	Fixed		
Age 75+	99,307	Fixed		
Cost of dialysis session (Medicare)	265.57	Fixed		^42^
Cost of hospitalization	17,226 (16,160−17,226)	Gamma(k,θ)	*k* = 1002, *θ* = 18.23	^31,32^
Annual discount rate	3% (2%−9%)	Fixed		^40^

All costs are in U.S. dollars.

*AMT* anemia management tool, *CI* confidence interval, *Hb* hemoglobin, *HD* hemodialysis, *FFS* fee-for-service, *PSA* probabilistic sensitivity analysis.

**Table 3:** Input values and distributions used to calculate costs and savings from the use of erythropoiesis-stimulating agents (ESAs)

Input	Base case value (range)	Reference
ESA cost ($) per µg	1.3 (1.3−1.5)	Estimate
Median adult HD patient weight (kg)	81.4 (interquartile range: 68.1–98)	^11^
Reduction in mean ESA dose per 30 days (µg/kg)	0.4 (95% CI: −0.1–0.8)	^15^

We estimated the overall HR for mortality by exponentiating the HR per unit increase in Hb SD to the observed difference in SD. This calculation implies an exponential relationship between Hb variability and the risk of mortality and builds on the Cox proportional hazards (PH) model^29^. Because the 26-week Fuertinger et al.^15^ trial did not measure mortality (so trial-based PH diagnostics are unavailable) and PH diagnostics are not uniformly reported in the source cohorts, we treat proportional hazards as a working assumption. This is reasonable provided that the AMT produces a roughly stable difference in Hb variability over time; we assess uncertainty by varying both the HR per 1 g/dL and ΔSD in sensitivity analyses.

We calculated the rate of avoided hospitalization in the intervention group as follows. Fuertinger et al.^15^ demonstrated a hemoglobin SD reduction of 0.2 g/dL. To estimate the effect of this variability reduction on hospitalization risk, we derived the odds ratio (OR) for hospitalization by comparing the combined LAL (low amplitude, low frequency) and LAH (low amplitude, high frequency) groups—representing low variability—to the HA (high amplitude) group—representing high variability—as shown in Fig. 4 of Ebben et al.^7^. The OR is approximately 0.53, with a 95% confidence interval (CI) of 0.45 to 0.62.

Since the 0.2 g/dL reduction in SD reported by Fuertinger et al.^15^ is smaller than the difference in variability between the groups compared in Ebben et al.^7^ (see calculation below), we adjusted the OR accordingly. Using Chinn’s^30^ method, we approximated the SD difference corresponding to the observed OR of 0.53 and proportionally scaled the effect to reflect the 0.2 SD reduction achieved in Fuertinger et al.

Specifically, Chinn’s^30^ method translates ORs into standardized effect sizes (SMDs), approximating the number of SDs of variability reduction corresponding to the observed change in odds. Chinn states that the natural logarithm of an OR can be converted into an approximate difference in the Normal Equivalent Deviate (NED) by dividing by π/√3 (approximately 1.81). While NED is not identical to SMD, it serves as an intermediary in Chinn’s approximation, enabling the interpretation of ORs in terms of SD units.

With an OR of 0.53, the natural logarithm of the odds ratio is calculated as:

2 $$\mathrm{ln}(0.53)\approx -0.634.$$

Using Chinn’s conversion factor, we divided the natural logarithm of the odds ratio (−0.634) by 1.81 to approximate the SMD:

3 $$SMD=\frac{-0.634}{1.81}\approx -0.35.$$

This SMD corresponds to the effect of the variability difference between the “LAL and LAH” and “HA” groups. To adjust for a smaller variability reduction (0.2 SD instead of 0.35 SD), we scaled the effect size proportionally using the ratio:

4 $$\frac{0.2}{0.35}\approx 0.57.$$

We then raised the OR to this power, reflecting the scaled effect size for the 0.2 SD reduction:

5 $${\rm{Adjusted\; OR}}={0.53}^{0.57}\approx 0.70.$$

This result indicates that a 0.2 SD reduction in Hb variability is associated with a 30% decrease in the odds of hospitalization relative to the baseline high variability group. The adjusted 95% CI for the odds ratio, based on the 0.2 SD reduction, is approximately 0.63 to 0.76. This calculation is derived from the 95% CI comparing the combined “LAL and LAH” (low variability) group to the “HA” (high variability) group in Fig. 4 of Ebben et al.^7^.

We then converted the OR to a relative risk (RR), accounting for the baseline risk probability. The absolute reduction in hospitalizations was calculated by multiplying the RR with the all-cause annual hospitalization rate for adult hemodialysis patients (1.49 hospitalizations per year) as reported in Fig. 5.1a of USRDS^2^.

Hospitalization costs were sourced from a study on patients under 65 years of age with employer-sponsored insurance who initiated dialysis care^31^. These costs were adjusted based on the assumption that Medicare reimburses approximately 50% less for hospital services compared to private insurers^32^.

Regarding utility (QALY) weights for ESA-treated dialysis patients within versus outside the 10–11 g/dL range, we explored a small utility premium in sensitivity analysis. Specifically, we set Δ*u* = 0.005 (absolute units), an order of magnitude below typical EQ-5D minimal important differences (0.03–0.07). This premium was parameterized using trial-observed improvements^15^: the AMT arm achieved a +10 percentage-point median increase in the proportion of Hb values within target (95% CI: +3 to +16 pp), which translates to an incremental gain of ≈0.0005 QALYs per patient-year at Δ*u* = 0.005 (i.e., 0.005 × 0.10).

While virtually all dialysis patients aged 65 and older have Medicare coverage, we estimated the proportion of dialysis patients under 65 with Medicare as follows. A 2022 study on new dialysis patients aged 18–64 reported that, among those not enrolled in Medicare at initiation, enrollment by the end of the first year dropped to 58.5% in 2014–2016 from 73.1% in 2006–2010, providing the most direct estimate for nonelderly patients^33^. A 2020 study showed a broader decline in Medicare enrollment at outpatient dialysis facilities, from 88.5% in 2005 to 65.3% in 2016, reflecting a trend driven by younger patients due to increased private insurance uptake via the Affordable Care Act and the 2011 Medicare Prospective Payment System^34^. With non-Medicare coverage rising to 33.9% by 2016^34^ and no post-2016 data pinpointing under-65 enrollment, we extrapolated that the proportion of under-65 dialysis patients enrolled in Medicare likely trended slightly below 58.5%, yielding a 50–60% range as a realistic estimate for 2025. This estimate was varied in sensitivity analyses.

For ESA costs, we used the price of MIRCERA, the exclusive ESA used in the RCT by Fuertinger et al.^15^. Using MIRCERA in the model preserves the pharmacokinetic/pharmacodynamic assumptions and dosing cadence of the evidence base. MIRCERA, which is off-patent, is a pegylated form of erythropoietin designed to stimulate red blood cell production with an extended duration of action compared to other ESAs. However, instead of applying the official list price, we used a discounted price to account for price rebates from individual contracts. In the U.S., contracts with payers, such as insurance companies, Medicare, and pharmacy benefit managers, typically include confidential price rebates. These discounts are influenced by factors such as the volume of use, payer size, and competitive market conditions.

For our calculations of ESA costs and savings, we assumed an average adult HD patient weight of 81 kg in the base case and applied the reduction in mean ESA dose associated with AMT, as reported by Fuertinger et al.^15^ (Table 2).

In the base case, we assumed the dialysis center does not currently have CLM and would need to make an initial investment in CLM, CLM consumables, and AMT. The costs included the AMT fee (29 to 41 cents per treatment), ongoing consumable costs for CLM ($1.35 to $2.00 per session), and the depreciated investment in the Crit-Line device (49 cents per session). The device cost was based on an assumption that one Crit-Line device is shared by three dialysis machines, with the device depreciated over a 10-year period. This results in an effective usage equivalent to approximately 3.3 years per device when distributed across machines.

In a sensitivity analysis, we assumed that the dialysis center has already invested in CLM but is not using it for all patients. In this case, the costs would include the purchase of bloodlines and AMT. Alternatively, we assumed the dialysis center has fully implemented Crit-Line for all patients and already has the necessary bloodlines, so only the costs of AMT would apply.

By utilizing AMT to reduce a higher-than-expected 30-day readmission rate to an average level, we assumed that each dialysis patient could avoid a 0.12% reduction in Medicare payments, as stipulated by the CMS QIP. This figure represents the average QIP payment reduction for large ESKD facilities^35^.

To estimate the total profit for a representative U.S. dialysis center, the center size was derived from publicly available data provided by major U.S. dialysis providers^36–39^, yielding an average of approximately 70 patients per center.

In the base case Markov model analysis, an annual discount rate of 3% was applied to both costs and effects, aligning with the recommendations of Sanders et al.^40^. Sensitivity analyses explored alternative discount rates of 2% and 9%.

All cost figures were adjusted to 2022 U.S. dollars using the U.S. Consumer Price Index for medical care services.

The study conducted one-way deterministic sensitivity analyses to evaluate the effects of upper and lower bounds for each uncertain variable. This approach varied one parameter at a time while keeping all others constant, enabling an assessment of each variable’s impact on the overall results. Table 2 lists the ranges used. In addition to one-way analyses, we performed a PSA using MCS with 1000 iterations to examine how simultaneous changes in multiple variables affect the NMB of AMT.

As shown in Table 2, utilities were modeled with a beta distribution because their values are confined between 0 and 1. Relative risks and hazard ratios were assumed to follow a normal distribution after logarithmic transformation, while hospitalization costs were modeled with a gamma distribution. The difference in Hb standard deviation was modeled with a Beta-PERT distribution, which respects the bounded support and observed right-skew. Due to the lack of detailed distribution data (e.g., confidence intervals), the proportion of dialysis patients under age 65 with Medicare coverage was modeled using uniform distributions.

Patient weight, which influences ESA dosing calculations, was modeled using a log-normal distribution. This choice reflects the non-negative, right-skewed nature of body weight data and was based on reported median and interquartile range values^11^.

The reduction in ESA dose associated with AMT was modeled using a normal distribution.

Box 1 Assumption Box—Key modeling assumptions (with justifications)

- Model structure: Two states (alive/dead); hospitalizations modeled as events. *Parsimonious structure; captures outcomes driving costs/QALYs*.
- Hb “in-range” utility: no base-case premium; scenario with small premium (Δ*u* = 0.005). *Prior modeling found no meaningful difference; scenario confirms robustness*.
- AMT effect on Hb stability: sustained reduction in intrapatient Hb SD as in the RCT. *Implements observed practice change*.
- Mortality mapping: log-linear mapping from Hb SD using HR per 1 g/dL; PH treated as a working assumption. *Cox-based evidence; PH not testable in 26-week trial—handled in sensitivity analyses*.
- Hospitalization risk: variability–utilization evidence scaled to the trial’s ΔSD. *Aligns effect size with observed stability change*.
- ESA agent: MIRCERA exclusively (prices/dosing). *RCT/controller are MIRCERA-specific; pricing/dose varied in scenarios*.
- WTP proxy: HD vs. “no KRT” as conservative lower bound; CPM/palliative implies higher WTP. *Real-world comparators have non-zero costs/benefits*.
- Discounting & price year: 3% for costs/effects; common inflation year. *Standard U.S. practice*.
- Population: average Medicare in-center HD baseline (USRDS). *Matches the dominant U.S. setting and trial context*.

## Data Availability

All data generated or analyzed during this study are included in this published article.
